# Cork Oak Endophytic Fungi as Potential Biocontrol Agents against *Biscogniauxia mediterranea* and *Diplodia corticola*

**DOI:** 10.3390/jof6040287

**Published:** 2020-11-14

**Authors:** Daniela Costa, Rui M. Tavares, Paula Baptista, Teresa Lino-Neto

**Affiliations:** 1 BioSystems & Integrative Sciences Institute (BioISI), Plant Functional Biology Centre, University of Minho, Campus de Gualtar, 4710-057 Braga, Portugal; danielacosta@bio.uminho.pt (D.C.); tavares@bio.uminho.pt (R.M.T.); 2 Centro de Investigação de Montanha (CIMO), Instituto Politécnico de Bragança, Campus de Santa Apolónia, 5300-253 Bragança, Portugal; pbaptista@ipb.pt

**Keywords:** fungi, endophytes, *Biscogniauxia mediterranea*, *Diplodia corticola*, biocontrol, cork oak

## Abstract

An increase in cork oak diseases caused by *Biscogniauxia mediterranea* and *Diplodia corticola* has been reported in the last decade. Due to the high socio-economic and ecologic importance of this plant species in the Mediterranean Basin, the search for preventive or treatment measures to control these diseases is an urgent need. Fungal endophytes were recovered from cork oak trees with different disease severity levels, using culture-dependent methods. The results showed a higher number of potential pathogens than beneficial fungi such as cork oak endophytes, even in healthy plants. The antagonist potential of a selection of eight cork oak fungal endophytes was tested against *B. mediterranea* and *D. corticola* by dual-plate assays. The tested endophytes were more efficient in inhibiting *D. corticola* than *B. mediterranea* growth, but *Simplicillium aogashimaense, Fimetariella rabenhorstii, Chaetomium* sp. and *Alternaria alternata* revealed a high potential to inhibit the growth of both. *Simplicillium aogashimaense* caused macroscopic and microscopic mycelial/hyphal deformations and presented promising results in controlling both phytopathogens’ growth in vitro. The evaluation of the antagonistic potential of non-volatile and volatile compounds also revealed that *A. alternata* compounds could be further explored for inhibiting both pathogens. These findings provide valuable knowledge that can be further explored in in vivo assays to find a suitable biocontrol agent for these cork oak diseases.

## 1. Introduction

Cork oak (*Quercus suber* L.) is an evergreen tree species that covers between 1.7 and 2.7 million ha in the western Mediterranean, distributed among Portugal, Spain, France, Italy, Morocco, Tunisia and Algeria [1]. Cork oak forests display a high socio-economic and ecological importance, being mainly explored for cork production [2]. The synthesis of cork, a homogeneous tissue of phellem cells, depends on the activity of the cork cambium, which covers the trunk and branches of cork oak. Due to the interesting and unique set of physical, biological and chemical properties, such as sealing and insulating features, cork is mainly used for the production of bottle stoppers [3]. Every year, 80% of worldwide produced cork comes from the Iberian Peninsula, in which Portugal is responsible for almost half of the total global production [2,4].

In recent years, cork oak forests have been declining in the Mediterranean region, which will be further enhanced by the combined effect of global warming and drought [5]. Indeed, the climate change predictions reveal the Mediterranean region as one of the most affected regions, and Mediterranean forests as one of the most vulnerable ecosystems to the combined effect of temperature increase and precipitation decrease [6,7]. Not only the geographic distribution of plant pathogens is predicted to be reshuffled [8], but also the severity of plant diseases and the rate at which endophytes switch from mutualistic to pathogenic behavior are expected to increase [9,10]. Indeed, during the last decades, an increase in cork oak diseases has been reported [11], including charcoal disease (caused by *Biscogniauxia mediterranea* (De Not.) Kuntze; Xylariales) and bot canker (caused by *Diplodia corticola* A.J.L. Phillips, A. Alves and J. Luque; Botryosphaeriales). Both pathogenic fungi have an endophytic lifestyle and opportunistic behavior, which account for the increase in diseases incidence in cork oak trees under environmental stress [12]. These diseases cause high economic losses due to the development of symptoms in cork oak trunk that affect both cork production and quality, and eventually lead to tree death [13].

Few preventive measures for cork oak charcoal disease and bot canker are currently known. Good phytosanitary practices in cork oak forests are essential to prevent the spreading of diseases through spore release and colonization from tree wounds derived from pruning or cork extraction [11]. Thiophanate-methyl and carbedazim fungicides have been found effective as a preventive measure against *D. corticola* [14,15]. However, the application of fungicides has been increasingly restricted due to the potential negative impact on human health and the environment. Therefore, more environmentally friendly strategies, such as the use of biological control agents, are now being explored for controlling many forest tree diseases [16,17]. Indeed, promising results have been obtained for restricting cork oak diseases. For example, the use of *Fusarium tricinctum* resulted in a reduced mortality of *Q. cerris* and *Q. pubescens* seedlings inoculated with *D. corticola* [18]. Further, *Trichoderma* spp. presented a high in vitro antagonistic potential against *B. mediterranea* and *D. corticola* [19]. In particular, a cork oak endophytic strain of *Trichoderma citrinoviride* produces peptaibols capable of inhibiting cork oak pathogens’ in vitro growth [20]. Spray suspensions of *T. atroviride* and *T. viride* combined with sanitation and scarification practices were recently suggested for decreasing charcoal disease incidence in *Q. castaneifolia* trees [21]. In the present study, we hypothesize that cork oak fungal endophytes are able to control cork oak pathogens’ (*B. mediterranea* and *D. corticola*) growth and we intend to highlight the most promising cork oak endophytes to be used as a biocontrol strategy. Specifically, we aimed to identify endophytic fungal species of cork oak that can be used to control *B. mediterranea* and *D. corticola*, by studying their antifungal activities against both pathogens and by characterizing the interactions between endophytes and pathogens.

## 2. Materials and Methods

### 2.1. Cork Oak Forests Sampling and Endophyte Recovery

**Figure 1:**
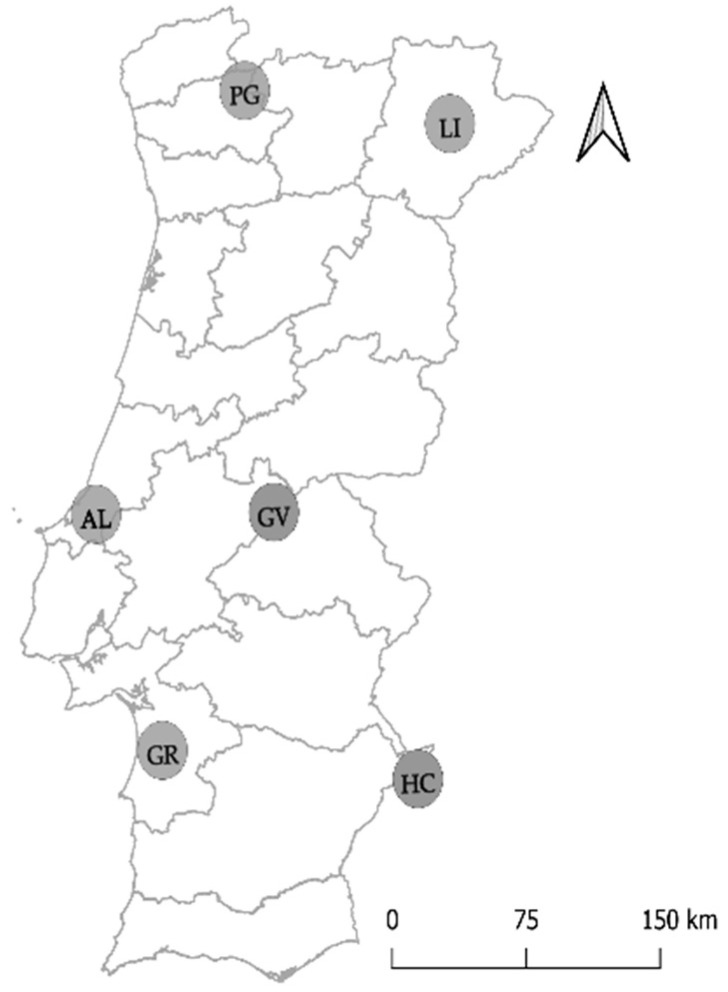
Distribution of cork oak stands sampled in Portugal. Letters represent sampled location (PG: Peneda-Gerês; LI: Limãos; GV: Gavião; AL: Alcobaça; GR: Grândola; HC: Herdade Contenda).

Cork oak samples were collected from eight different forests in Portugal (Table S1). Two forests were sampled from the National Park of Peneda-Gerês (PG-ER and PG-RC) and Herdade da Contenda (HC-CT and HC-MA). A single forest was sampled from Limãos (LI), Alcobaça (AL), Gavião (GV) and Grândola (GR). Between April and October of 2017, five to six trees were sampled from each cork oak forest, considering trees at different disease severity levels (Table 1; Figure 1). Disease severity levels were grouped into three categories and determined by considering defoliation (5 levels: 0–10%—no damage; 11–25%—light damage; 26–50%—moderate damage; 51–90%—severe damage; > 91%—extreme damage), as well as canopy and trunk damages (3 levels: 0—no damage; 1—moderate damage; 2—severe damage), for different factors (dried, wilting and decolorated leaves, presence of cankers, decolorated trunk, presence of exudates and visible sporulation). Trees were considered as healthy, presenting mild symptoms or declining. Healthy trees presented no or light defoliation, displaying low canopy and trunk damage (maximum of two factors with 1 damage level, but not affecting overall vigor of the tree). When tree vigor was moderately affected by a combination of factors (moderate defoliation and/or some canopy and trunk damage), trees were considered as displaying mild symptoms. Declining trees presented a clear decline in their vigor (accentuated or very accentuated defoliation that could be coupled with more than three factors classified with 1 and/or 2 damage levels).

Five to seven branches were collected from each cork oak tree. Twigs were collected from each branch and thoroughly washed in tap water. To obtain only endophytes, a surface sterilization was performed based on the method described by Martins et al. [22]. Twigs were sequentially immersed in ethanol 70% (*v*/*v*) for 2 min, bleach (3–5% chlorine) for 6 min and ethanol 70% (*v*/*v*) for 1 min, followed by three washes in sterile deionized water (1 min each) and drying. Sterile twigs were cut into segments (4–5 cm) and transferred to Potato Dextrose Agar (PDA) medium (5 segments/plate). Three replicates were used for each branch. Sterilization controls were performed by spreading the last washing water (10 μL) onto PDA medium. Incubation was performed in the dark, at room temperature (21–23 °C). The outgrowing fungi were recognized as endophytic fungi and were successively subcultured in fresh PDA medium until pure cultures were obtained.

### 2.2. Endophytic Fungi Identification and Selection of Potential Antagonistic Fungi

Endophytic fungi were grouped into morphotypes, according to their cultural features (color, shape, elevation and margins) [23]. From each morphotype, at least three isolates were used for DNA extraction, using the Quick-DNA Fungal/Bacterial Miniprep Kit (Zymo Research, Irvine, CA, USA). The fungal rDNA-ITS region was amplified using universal primer pairs *ITS1F* (5’-CTTGGTCATTTAGAGGAAGTAA-3’) and *ITS2* (5’-GCTGCGTTCTTCATCGATGC-3’), or *ITS1F* and *ITS4* (5’-TCCTCCGCTTATTGATATGC-3’) [24]. PCR mixtures (25 µL) contained 1× Complete NH_4_ Reaction Buffer (BIORON GmbH, Germany), 200 µM of each dNTP (NZYTech, Portugal), 1 µM of each primer, 1 μL of DNA template and 1.25 U of DFS-Taq DNA Polymerase (BIORON GmbH, Germany). Amplifications were performed using the following protocol: initial denaturation 5 min at 94 °C; 35 cycles of 30 s at 94 °C, 30 s at 52 °C (or 54 °C with *ITS1F*-*ITS4*) and 60 s at 72 °C; final elongation at 72 °C for 10 min. PCR products were run on a 1% (*w/v*) agarose gel, stained with Green Safe Premium (NZYTech, Portugal). PCR products were purified using isopropanol 75% (*v*/*v*) and sequenced by Macrogen, Inc services (Madrid, Spain). DNA sequences were trimmed by sequencing quality and alignments were performed in Geneious 2010.4.8.5 (https://www.geneious.com). All sequences were identified using the UNITE [25] and NCBI GenBank databases and taxonomic classification was assigned for those with a similarity higher than 97%. Alignments with NCBI deposited sequences were used to confirm taxonomic classification and to attempt taxonomic classification of those sequences with similarity between 80 and 97%. Fungal identifications were supported by cultural and morphological features of fungal cultures. Fungal sequences were deposited in GenBank (www.ncbi.nlm.nih.gov/genbank/) under accession numbers MT819608–MT819946.

For understanding the ecological roles of fungal communities, the identified fungi were categorized into functional groups (pathogenic and/or beneficial), based on the bibliographic research [26,27]. Identified operational taxonomic units (OTUs) were assigned as beneficial when described as promoting plant growth and/or protecting the host against biotic or abiotic stress, while the pathogenic group contained fungi causing disease to its host and latent pathogens. Fungi belonging to other functional groups (mutualism, commensalism, etc.) were assigned to other groups and those with unknown function to unknown. Fungi belonging to more than one group (for example, pathogenic–beneficial) were added to both groups. For proceeding to the antagonism assays, endophytes were selected based on their potential beneficial role and availability. All groups were considered with the exception of those fungi exclusively described as pathogenic and all cork oak pathogens. Eight fungal isolates were selected to be tested against the pathogens in antagonistic assays due to their cultural readiness, namely *Simplicillium aogashimaense* isolate Gr67, *Coniothyrium carteri* isolate Gv5, *Diaporthe passiflorae* isolate Erm6, *Fimetariella rabenhorstii* isolate Br33, *Fusarium oxysporum* isolate Cab77, *Chaetomium* sp. isolate Erm52, *Alternaria alternata* isolate Cab37 and *Penicillium olsonii* isolate Gv63. *B. mediterranea* and *D. corticola* isolates were inoculated on cork oak stems to confirm symptoms development, completing Koch’s postulates. 

### 2.3. Antagonistic Assay In Vitro by Dual-Plate and Categorization of Fungal Interactions

The eight selected endophytic fungi were tested in vitro against phytopathogens *Biscogniauxia mediterranea* isolate Gr13 and *Diplodia corticola* isolate Gr23, both isolated from cork oak trees showing mild symptoms. Antagonistic assays were performed by the dual-culture method in PDA medium. Fungal plugs (5 mm) of an actively growing endophyte and pathogen were placed 3 cm apart from each other (using 9 cm diameter Petri dishes) and incubated in the dark, at 28 °C (±2 °C). Those endophytes displaying a slow growth rate (<0.1 cm^2^/h; Table S2) were inoculated 72 h before the pathogen. In parallel, control plates were similarly prepared but using a single pathogen (or endophyte) plug. All plates were photographed 72 h after being inoculated with the endophyte and/or pathogen, and growth area was measured using *ImageJ 1.50i* software [28]. Growth areas were used to determine the percentage of mycelial growth inhibition, according to the formula: (Ac−Ai)/Ac × 100, where Ac is the area of fungal growth in the control plate and Ai is the area of fungal growth with the interacting fungus. Three independent experiments were performed (with at least 3 replicates each) for all endophyte/pathogen combinations. Statistical analysis was performed using ANOVA in GraphPad Prism 7.00 software (La Jolla, CA, USA) to determine the impact of endophytes on pathogen growth.

Fungal interactions between endophytes and pathogens were categorized based on Tuininga [29]. Considering that (-) corresponds to mycelial growth decrease, (+) to growth increase and (0) to similar mycelial growth, interactions among endophyte/pathogen could be defined as co-antagonism (-/-), antagonism (-/0), agonism (-/+), co-habitation (0/0), commensalism (0/+) and mutualism (+/+). For describing the fungal interactions, dual-culture plates were observed daily for 15 days. Mycelial interactions were also recorded based on Badalyan et al. [30], who defined the following classes: A—deadlock with mycelial contact; B—deadlock at distance; C—replacement, overgrowth without deadlock; CA1 and CA2—partial and complete replacement after initial deadlock with mycelial contact; and CB1 and CB2—partial and complete replacement after initial deadlock at a distance. Finally, hyphal interactions were observed using mycelia from the interacting region of dual-culture plates, after 15 days of interaction, and compared with mycelia taken from controls. Photographs were taken using a Leica MC170 HD digital camera attached to a Leica S9 D stereomicroscope (Leica Microsystems, Germany) or using an automated Leica DM5000B microscope (Leica Microsystems, Germany).

### 2.4. Antifungal Non-Volatile Compounds Assay

The antagonistic effect of the metabolites produced by the eight selected fungal endophytes was tested using a method adapted from Campanile et al. [18]. Three mycelial fungal plugs (5 mm) of actively growing endophytes were inoculated in Erlenmeyer flasks (250 mL) containing 50 mL of sterile Potato Dextrose Broth (BD Difco™, Switzerland). Culture flasks were placed in an orbital incubator at 28 °C (±2 °C), with 150 rpm agitation, for 12 days. Liquid cultures were transferred to falcon tubes and centrifuged for 15 min at 12,000 rpm. The supernatant was collected and filtered through a 0.22 µM membrane filter to remove hyphal residues and conidia. Fungal filtrates were added to sterile warm PDA medium [20% (*v*/*v*)] and poured into 9 cm Petri dishes. A mycelial plug of an actively growing pathogen (*B. mediterranea* or *D. corticola*) was placed in the center of the plate. Control plates were similarly prepared but containing PDA without a fungal filtrate. Mycelial growth areas were determined as previously referred and mycelial growth inhibition was determined according to the formula: (Ac−Af)/Ac × 100, where Ac is the area of fungal growth in the control plate and Af is the area of fungal growth in the presence of the filtrate. Three independent experiments were performed (with at least 3 replicates each) for all endophyte/pathogen combinations. Statistical analysis was performed using ANOVA in GraphPad Prism 7.00 software (La Jolla, CA, USA) to understand the impact of endophytic compounds on pathogen growth.

### 2.5. Antifungal Volatile Compounds Assay

The effect of volatile compounds produced by the eight selected fungal endophytes in inhibiting pathogen growth was evaluated by an inverted plate method [31]. For each endophyte/pathogen combination, two PDA plates were inoculated with a single mycelial plug (5 mm) of an actively growing endophyte (or pathogen). Then, the PDA plate with the pathogen was inverted on the top of the endophyte plate and both were sealed with parafilm. Incubation occurred at 28 °C (±2 °C), for 72 h. Control plates were performed without inoculation of the endophyte in the bottom plate. As previously described, those endophytes displaying a slow growth rate (<0.1 cm^2^/h; Table S2) were inoculated 72 h before the pathogen. The percentage of inhibition was calculated as mentioned before. Three independent experiments were performed (with at least 3 replicates each) for all endophyte/pathogen combinations. Statistical analysis was performed using ANOVA in GraphPad Prism 7.00 software (La Jolla, CA, USA) to understand the impact of endophytic volatiles on pathogen growth.

## 3. Results and Discussion

### 3.1. Endophytic Fungal Community of Cork Oak

Endophytic fungi of cork oak twigs were recovered from eight different forests in Portugal. From a total of 1117 fungal isolates, 440 were molecularly identified and grouped into 128 OTUs comprising 18 orders, 38 families, 45 genera and 39 species (Table 2). Only OTUs classified up to genus and species were considered in this work (70 OTUs). From these, and to the best of our knowledge, 54 fungal OTUs have never been reported as cork oak endophytes before (e.g., *Fimetariella rabenhorstii*, *Discosia* sp.), including 18 that have never been described as plant endophytes (e.g., *Caliciopsis beckhausii*, *Diaporthe passiflorae*, *Proliferodiscus* sp.) (Table 2). Among previously undescribed cork oak endophytes, 28 were only recovered from a single sampled cork oak forest, but others (26) were recovered from different locations, which strengthens their role as cork oak endophytes. For example, *Discosia* sp., *Cryphonectria naterciae* and *Neocucurbitaria* sp. were recovered from different Portuguese forests, or from forests displaying the highest (e.g., *F. rabenhorstii*) or the lowest (e.g., *Plectania rhytidia*) precipitation levels.

Some OTUs were more widespread throughout Portuguese cork oak forests than others. *B. mediterranea* was the only fungus identified in all cork oak stands and *Fusarium* sp. was present in all forests, with the exception of LI and GR. Further, *Penicillium* sp., *Sarocladium kiliense* and *Neocucurbitaria* sp. were recovered from almost all cork oak stands. In contrast, there were fungi (such as *Diplodia corticola*, *Nonappendiculata quercina*, *Ciboria* sp. and *Pezicula cinnamomea*) that were only recovered from one sampling location. Since many fungi are difficult to be cultured, culture-dependent methods are known to underestimate fungal communities [32]. The used approach only targeted those endophyte fungi that can be easily cultured on artificial media and have a rapid growth rate. Furthermore, as many endophytes do not sporulate in culture, their morphotype discrimination through cultural features is challenging and might have been underestimated. For these reasons, we are aware that the performed endophyte survey through cultural methods (and based on morphotypes discrimination) misrepresented the endophyte diversity in cork oak forests, as already reported elsewhere for other plant hosts [32]. Accordingly, our study reported a strong dominance of fungi belonging to Ascomycota (only one OTU belonged to the Basidiomycota phylum), like previously reported in other studies using culture-dependent methods, e.g., [33,34]. Culture-independent methods could have provided a different picture of fungal communities. For example, in grapevine, besides Ascomycota (described using cultural methods), Basidiomycota and Zygomycota fungi were additionally detected recurring to a metabarcoding approach [33]. Despite the recognized limitations, the used culture-dependent approach provided the availability of endophyte isolates to proceed in searching for biocontrol strategies.

**Figure 2:**
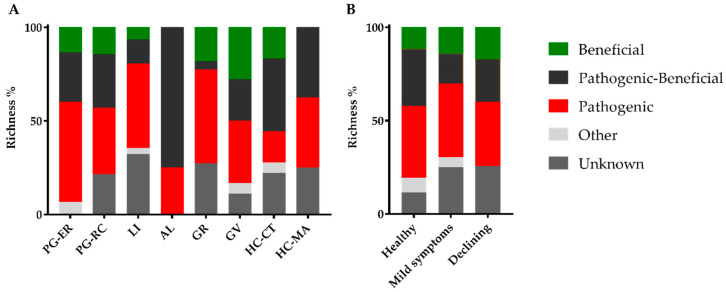
Cork oak endophytic fungal functional groups presented in cork oak forests (**A**) and disease severity level (**B**). Operational taxonomic units (OTUs) belonging to more than one functional group were added to both, except for the pathogenic–beneficial group that is represented as such.

**Figure 3:**
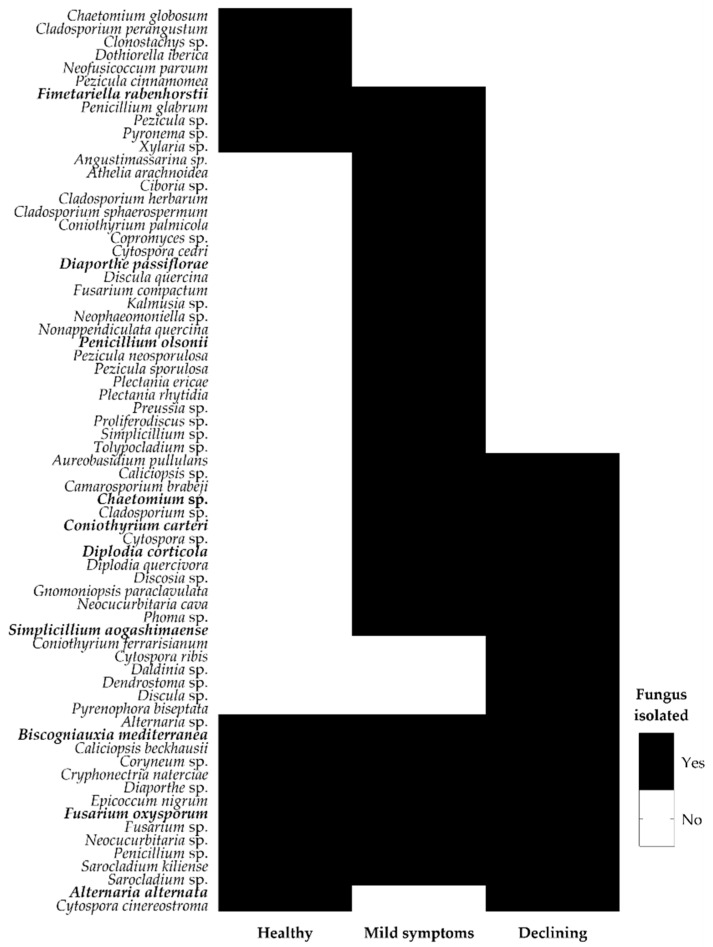
Endophytic fungal OTUs recovered from cork oak trees at different disease severity levels. Black/white color indicates fungal isolation/no isolation from trees with different disease severity levels. Isolates used in this work are depicted in bold.

The ecological role of each identified fungal species was determined based on the literature. As the functional role of certain endophytes could change according to their plant host genotype, during different stages of the plant life cycle or in extreme conditions [26], many endophytes were included in mixed groups (such as the pathogenic–beneficial group). Furthermore, within certain genera, there are species known to be pathogenic and others beneficial, being impossible to consider a single functional role. Despite these constrains, the number of identified OTUs displaying a phytopathogenic role was higher, when compared to other functional groups (Table 2). A total of 39 OTUs were considered as displaying a phytopathogenic role (including exclusive phytopatogenic (26), phytopathogenic–beneficial (10) and phytopathogenic–other (3) functional groups), while only 21 displayed a beneficial role (including beneficial (10), phytopathogenic–beneficial (10) and beneficial–other (1) functional groups). The richness of fungi displaying a potential phytopathogenic role was also higher than any other functional group in all cork oak stands and whatever the cork oak tree disease severity level (Figure 2). Among them, few cork oak pathogens were found: *B. mediterranea* [12], *Coryneum* sp. [35], *Cryphonectria naterciae* [36], *D. corticola* [37], *D. quercivora* [38], *Discula quercina* [39] and *Neofusicoccum parvum* [35]. Interestingly, *B. mediterranea*, *Coryneum* sp. and *C. naterciae* were isolated from trees in all disease severity levels, while *D. corticola*, *D. quercivora* and *Discula quercina* were only isolated from trees with declining symptoms (Figure 3). In agreement, cork oak pathogens (*B.mediterranea* and *Coryneum* sp.) were isolated from healthy cork oak trees in Italy [12,40]. The presence of *D. corticola*, *D. quercivora* and *Discula quercina* in declining cork oak trees agrees with their role as emerging pathogens to *Quercus* spp. in different regions of the world [11,41], which is emphasized by their risk to cork oak health as previously reported [39,42]. Concerning the fungi displaying a beneficial role, AL forest presented the highest number of OTUs with a potential beneficial role (all described as pathogenic–beneficial), but GV forest was the richest with exclusive beneficial fungi (Figure 2A). GV forest corresponded to one of the forests displaying the highest number of declining trees (results not presented), which agrees with the finding that trees with declining symptoms displayed a higher richness of exclusive beneficial fungi (a non-significant 1.5-fold increase in relation to healthy trees; Figure 2B). The role of stress-affected plants in recruiting beneficial microorganisms is still under debate and a “cry-for-help” hypothesis was recently proposed, in which plants are able to recruit plant-protective microbes when they are under attack by pathogens [43,44]. For example, tomato plants under stress produce root exudates to signal the beneficial *Trichoderma harzianum* T22 strain to direct growth toward the plant host [45]. However, further studies on endophyte distribution among healthy and diseased cork oak trees are still needed for providing clear evidence that support a “cry-for-help” strategy for the sustainability of threatened cork oak forests.

### 3.2. Interactions of Endophytes against B. mediterranea and D. corticola

**Figure 4:**
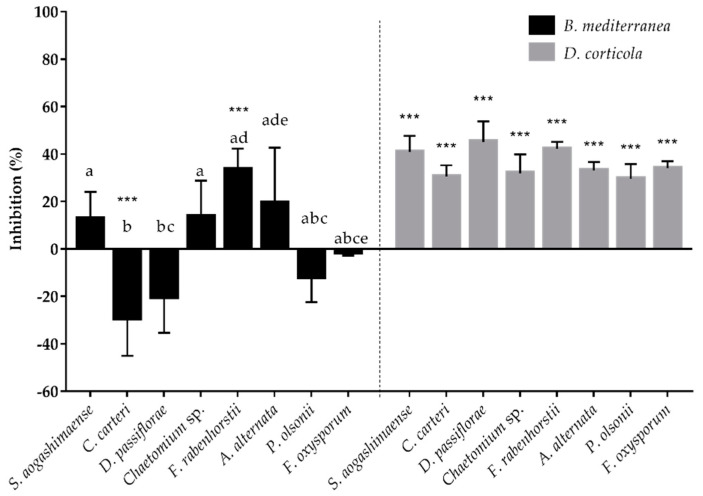
Inhibition of *B. mediterranea* and *D. corticola* growth caused by the tested endophytic fungi in dual-culture assay. Negative values indicate that the area of the plate covered by the interacting pathogen was higher than that of the control. Different letters represent statistical significance (*p ≤* 0.005) between endophytes in each antagonistic assay and *** (*p ≤* 0.001) represents statistical significance between the control and co-culture of a pathogen.

Eight endophyte isolates were selected for testing their ability to inhibit *B. mediterranea* and *D. corticola* growth, based on their potential beneficial ecological role, availability and culture readiness. *D. corticola* mycelial growth was persistently inhibited by the presence of these cork oak endophytes, while *B. mediterranea* was differentially inhibited by endophytes (Figure 4). Dual-plate experiments revealed that *B. mediterranea* growth was inhibited by *F. rabenhorstii* (33.4%, *p ≤* 0.001), *A. alternata* (19.3%), *Chaetomium* sp. (13.6%) and *S. aogashimaense* (12.6%), whereas the other fungal endophytes promoted or did not affect pathogen growth (Figure 4). Regarding endophytes inhibiting *B. mediterranea* and considering the effect of pathogens on endophyte growth (Figure S1), *S. aogashimaense* was the only endophyte not inhibited by this pathogen, displaying a typical antagonist interaction (0/-) (Table 3). Although *F. rabenhorstii* strongly inhibited *B. mediterranea*, there was a negative impact of the pathogen on this endophyte growth (co-antagonism; -/-). Both endophytes displayed a similar interaction with the *D. corticola* pathogen, resulting in antagonism/co-antagonism with pathogen inhibition (40.8%, *p ≤* 0.001 by *S. aogashimaense*; 42.2%, *p ≤* 0.001 by *F. rabenhorstii*). In contrast, *A. alternata* and *Chaetomium* sp. increased their growth while inhibiting *B. mediterranea*, thus displaying agonist interactions (+/-) with this pathogen. Both endophytes displayed a distinct interaction with *D. corticola*. *A. alternata* was not affected (antagonism; 0/-) and *Chaetomium* sp. was inhibited by this pathogen (co-antagonism; ­/­). Except for *F. oxysporum* (antagonism; 0/-), all the other tested endophytes also revealed a co-antagonism interaction with *D. corticola*.

**Figure 5:**
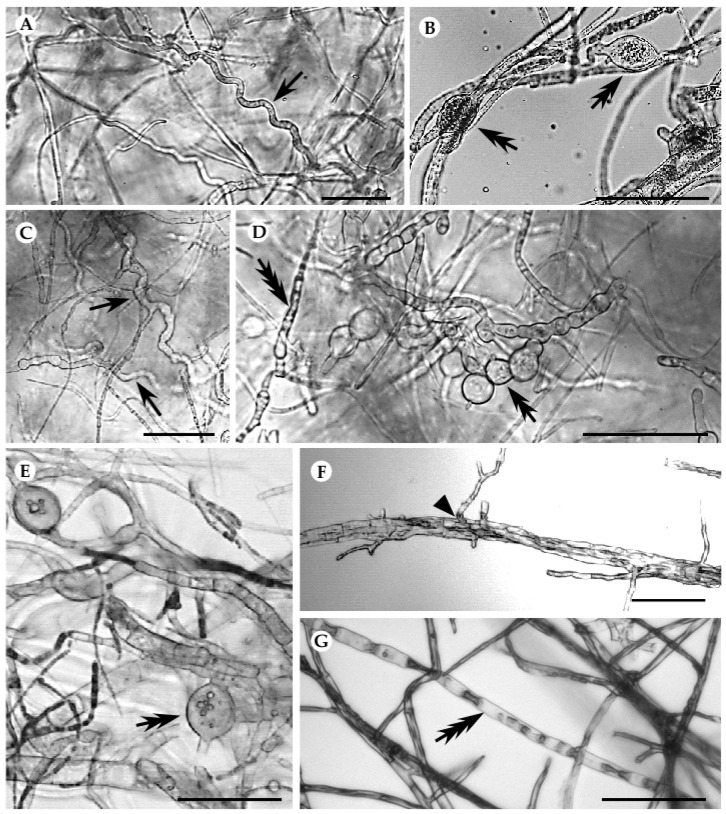
Hyphal modifications produced in the interaction between *S. aogashimaense* and *B. mediterranea* (**A**,**B**), *S. aogashimaense* and *D. corticola* (**C**,**D**) and *A. alternata* and *B. mediterranea* (**E**–**G**). Single arrows designate coiled hyphae, double arrows vesicle-like structures and triple arrows hyphal vacuolization. Black line represents 50 µM scale.

Fungal interactions were further evaluated by following the macro- and microscopic modifications of mycelia in the interaction region. When interacting with both pathogens, *S. aogashimaense* revealed a deadlock at distance (B) interaction type (Figure S2A,B) and caused visible modifications on hyphae of both pathogens (Figure 5A–D). In the presence of this endophyte, *B. mediterranea* showed typical hyphal deformations caused by the interacting partner, such as the presence of coiled hyphae and production of vesicle-like structures (Figure 5A,B). Further, *D. corticola* suffered mycelial modifications caused by interaction with *S. aogashimaense*, which included hyphal coiling and vacuolization, as well as the production of vesicle-like structures (Figure 5C,D). Such alterations have been frequently reported in different incompatibility systems [128,129] and have been related to programmed cell death (PCD) events occurring during interaction [130]. The *A. alternata–B. mediterranea* interaction also revealed hyphal deformations, such as hyphal vacuolization, production of vesicle-like structures and hyphal penetration that resemble a mycoparasitism interaction (Figure 5E–G). Accordingly, instead of a deadlock at distance interaction type, in this interaction, there was a partial replacement of mycelia after an initial deadlock with mycelial contact (CA1; Table 3 and Figure S2C). Interestingly, although the inhibitory activity of an *A. alternata* isolate from *Q. cerris* against *D. corticola* has been reported, both in dual-culture and in planta [18], we have not detected hyphal distortions in the *A. alternata–D. corticola* interaction. All the other studied interactions did not reveal hyphal distortions, even though similar interaction types have been detected after 15 days of interaction (partial replacement after initial deadlock with mycelial contact; CA1), as well as a deadlock with mycelial contact (A) (Table 3).

### 3.3. Fungal Inhibitors Production by Cork Oak Endophytes

**Figure 6:**
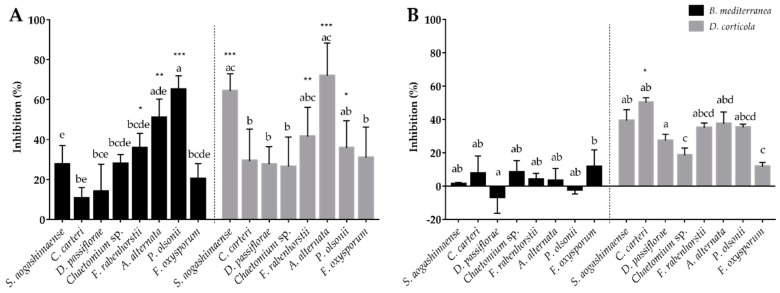
Inhibition of *B. mediterranea* and *D. corticola* growth caused by non-volatile (**A**) and volatile compounds (**B**) produced by the tested endophytic fungi. Negative values indicate that the area of the plate covered by the interacting pathogen was higher than that of the control. Different letters represent statistical significance (*p* < 0.005) between endophytes in each antagonistic assay and * (*p* ≤ 0.05), ** (*p ≤* 0.01) and *** (*p ≤* 0.001) represent statistical significance between the control and co-culture of a pathogen.

For understanding the production of inhibitors by the tested endophytes, their non-volatile and soluble compounds (produced in liquid culture) and volatile emissions were tested against *B. mediterranea* and *D. corticola* pathogens. Endophytic soluble compounds seem to have more impact on inhibiting mycelial growth than volatile compounds, mainly against *B. mediterranea* (Figure 6). However, this primary conclusion should be taken with some precaution as these inhibitory compounds were tested by using different strategies for their collection and assay. The endophytes that revealed the greatest inhibitory activity against *B. mediterranea* in the dual-culture method (*F. rabenhorstii*, *A. alternata*, *Chaetomium* sp. and *S. aogashimaense*) produced a soluble extract with high anti-fungal activity against both pathogens. These endophytes inhibited from 28% to 51% of *B. mediterranea* growth (*F. rabenhorstii p ≤* 0.05 and *A. alternata p ≤* 0.01) and from 26% to 72% of *D. corticola* growth (*S. aogashimaense* and *A. alternata p ≤* 0.001; *F. rabenhorstii p ≤* 0.01). The volatile emissions from the same endophytes also revealed inhibitory activities against *B. mediterranea* and *D. corticola* (ranging from 1% to 8% for *B. mediterranea*, and 18% to 39% for *D. corticola*). Multiple studies have already revealed the antifungal activity of genera from tested endophytes, in particular of *Chaetomium* spp., e.g., [131], *Coniothyrium* spp., e.g., [132], *Diaporthe* spp., e.g., [133], *Penicillium* spp., e.g., [134], and *Simplicillium* spp., e.g., [135], as well as their ability to produce a battery of antifungal compounds. However, less information is available on the inhibitory activity of *Fimetariella* spp. or the antifungal activities of *A. alternaria* and *F. oxysporum* species, which have been mainly recognized as important plant pathogens. This work provides new information related to the production of antimicrobial compounds, mainly from *F. rabenhorstii* and *A. alternata*, against cork oak pathogens. 

Interestingly, endophytes with no inhibitory activity against *B. mediterranea* in dual-plate methods also revealed the production of inhibitors for this phytopathogen. For example, the soluble compounds of *P. olsonii* revealed the highest inhibition (65%, *p ≤* 0.001) of all against *B. mediterranea*, and volatiles from *C. carteri* and *F. oxysporum* also exhibited high inhibitory activity against the same pathogen (8% and 12%, respectively). These results suggest that the inhibitory effect of a specific fungal isolate against a phytopathogen may be due to the production of multiple compounds that could act in a synergistic or antagonistic way [136]. Accordingly, the production of inhibitory compounds such as volatiles, antibiotics and other secondary metabolites is gaining biotechnological interest for the control of phytopathogens. For example, the potential of *S. coffeanum* volatile compounds against *Aspergillus* species was reported by Gomes et al. [95] and *S. lamellicola* was used to produce a fungicide against *Botrytis cinerea* [137]. The production of inhibitory compounds is gaining interest when produced by potential pathogens. For example, the potential of *Alternaria* sp. was already reported for the control of fungal and bacterial growth [103], but some species are widely known as phytopathogens [104], representing a disadvantage for field application. The same is described for the *F. oxysporum* phytopathogen [88,138]. Therefore, the recognition of non-volatile or volatile inhibitory compounds could represent a biotechnological advantage for using those isolates as biocontrol agents.

## 4. Conclusions

The use of naturally adapted endophytes (for a specific plant host/environment) in a biocontrol strategy has gained increasing interest for restricting plant diseases. In this cork oak endophyte survey, we detected a high number of fungal OTUs from trees displaying different disease severity levels. The number of OTUs belonging to the pathogenic functional group (including described pathogens to other plant species) was high, even when considering healthy trees. Isolates of potential pathogens (namely, *Alternaria alternata* and *Fusarium oxysporum*) have revealed a strong in vitro inhibitory effect against cork oak pathogens (*B. mediterranea* and *D. corticola*). In particular, an *A. alternata* isolate revealed a high inhibitory activity against both pathogens, promoting hyphal deformations on *B. mediterranea.* Although this was not the case when interacting with *D. corticola*, this pathogen was similarly inhibited by *A. alternata* and *F. oxysporum*, displaying a mycelial interaction type based on the partial replacement of mycelia after an initial deadlock with mycelial contact. Non-volatiles and volatiles obtained from these isolates (particularly from *A. alternata*) revealed inhibitory activity and their potential to be used in a biocontrol strategy for restraining cork oak diseases should be further explored. 

The colonization of plants by beneficial endophytes has been a useful biocontrol strategy. This work suggests *Simplicillium aogashimaense* as an antagonistic fungus towards *B. mediterranea* and *D. corticola* with potential to be used as a biocontrol agent against cork oak diseases. Indeed, *S. aogashimaense* presented promising results inhibiting both pathogens’ growth, which was reinforced by the promotion of pathogens’ hyphae deformations during interaction (deadlock at distance). A high ecological and economical value has been given to *Simplicillium* species due to their biocontrol role and production of bioactive compounds. For example, soybean plants when inoculated with *S. lanosoniveum* before infection with soybean rust pathogen (*Phakopsora pachyrhizi*) revealed reduced disease severity [139]. Although we believe *S. aogashimaense* could similarly be explored to control cork oak diseases, better understanding of its potential role as a biocontrol agent is still required. We thus conclude that cork oak endophytes could be further explored as biocontrol agents against cork oak diseases.

## Figures and Tables

**Table 1:** Collection of cork oak branches in sampled forests. The number of trees sampled from each disease severity level is presented. For more details of sampled forests, see Table S1.

Location	Cork Oak Stand	Collection Date	Disease Severity Level		
			Healthy	Mild Symptoms	Declining
Peneda-Gerês	PG-ER	May, 2017	2	3	0
	PG-RC	July, 2017	4	1	0
Limãos	LI	April, 2017	0	5	1
Gavião	GV	July, 2017	0	4	2
Alcobaça	AL	May, 2017	2	2	2
Grândola	GR	May, 2017	0	3	3
Herdade Contenda	HC-CT	October, 2017	2	2	2
	HC-MA	October, 2017	2	2	2

**Table 2:** Endophytic fungal OTUs recovered from twigs of cork oak trees located in different forests. Taxonomic classification (with closest match identity in brackets) and their respective functional group are revealed. Reports about their endophytic behavior are referred to. When existing, references related to cork oak are presented (bold). Isolates used in this work are depicted in red. Functional groups are represented as: P—pathogenic; B—beneficial; O—other; U—unknown.

Taxonomic Classification		Closest Match GenBank	Cork Oak Forests								Functional Group	Identified Endophyte
			PG-ER	PG-RC	LI	AL	GV	GR	HC-CT	HC-MA		
Ascomycota												
Amphisphaeriales	*Discosia* sp.	KU325138.1 (100%)			x				x		P [46]/B [47]	[48]
	*Nonappendiculata quercina*	MH554025.1 (98.78%)			x						U	-
Botryosphaeriales	*Diplodia corticola*	MT015621.1 (100%)						x			P [37]	**[40]**
	*Diplodia quercivora*	JX894205.1 (97.72%)						x			**P** **[38]**	**[11]**
	*Dothiorella iberica*	MT261024.1 (100%)							x		**P** **[49]**	**[12]**
	*Neofusicoccum parvum*	MT645697.1 (99.3%)	x								**P** **[35]**	[50]
Capnodiales	*Cladosporium herbarum*	LT854669.1 (99.22%)			x						P [51]	[52]
	*Cladosporium perangustum*	MK111614.1 (99.10%)								x	P [53]	[54]
	*Cladosporium* sp.	MN879328.1 (100%)			x			x			P [55]/B [56]	**[40]**
	*Cladosporium sphaerospermum*	MT645920.1 (99.51%)					x				B [57]	[57]
Coryneliales	*Caliciopsis beckhausii*	NR_132090.1 (99.57%)			x					x	U	-
	*Caliciopsis* sp.	NR_132090.1 (91.91%)			x			x			P [58]	-
Diaporthales	*Coryneum* sp.	MH674330.1 (95.54%)		x	x		x	x			P **[35]**	**[40]**
	*Cryphonectria naterciae*	MT645942.1 (100%)		x	x		x	x			P **[36]**	-
	*Cytospora cedri*	MN871816.1 (100%)			x			x	x		P [59]	-
	*Cytospora cinereostroma*	KY051964.1 (100%)						x		x	U	-
	*Cytospora ribis*	KP641138.1 (100%)			x						U	[60]
	*Cytospora* sp.	MK656248.1 (100%)			x			x			P [61]	**[40]**
	*Dendrostoma* sp.	MN447228.1 (99.66%)			x						P [62]	-
	*Diaporthe passiflorae*	NR_120155.1 (99.82%)	x								O [63]/P [64]	-
	*Diaporthe* sp.	MT561408.1 (99.48%)	x			x				x	P [65]	[65]
	*Discula quercina*	MH758705.1 (99.18%)			x						P **[39]**	**[40]**
	*Discula* sp.	KY367498.2 (94.23%)			x						P [66]	[67]
	*Gnomoniopsis paraclavulata*	MH863162.1 (100%)			x			x			U	-
Dothideales	*Aureobasidium pullulans*	MT645930.1 (99.57%)			x						O [68]/B [69]	**[40]**
Eurotiales	*Penicillium glabrum*	MT582777.1 (100%)		x			x	x			P [70]	[71]
	*Penicillium olsonii*	MT582783.1 (100%)					x				B [72]	[73]
	*Penicillium* sp.	LN901128.1 (99.54%)			x	x	x		x	x	O [74]/P [70]/B [72]	**[40]**
Helotiales	*Ciboria* sp.	KF545322.1 (94.59%)			x						P [75]	-
	*Pezicula cinnamomea*	MK907714.1 (100%)	x								P [76]	[77]
	*Pezicula neosporulosa*	KR859231.1 (100%)	x								P [78]	[79]
	*Pezicula sporulosa*	MH862573.1 (98.66%)					x				O[80]	[81]
	*Pezicula* sp.	MG098317.1 (100%)	x		x						O [82]/P [76]	[67]
	*Proliferodiscus* sp.	MN901941.1 (95.50%)			x						U	-
Hypocreales	*Clonostachys* sp.	MK789204.1 (91.84%)		x							B [83]	[84]
	*Fusarium compactum*	KJ562364.1 (98.53%)					x				P [85]	[86]
	*Fusarium oxysporum*	MT530243.1 (100%)	x	x		x			x		P [87]/B [88]	[89]
	*Fusarium* sp.	MT645120.1 (100%)	x	x		x	x		x	x	P [87]/B [18]	[18]
	*Sarocladium kiliense*	MK789203.1 (100%)	x			x	x		x	x	P [90]/B [91]	[92]
	*Sarocladium* sp.	MT645143.1 (99.36%)	x			x			x	x	P [93]/B [91]	[94]
	*Simplicillium aogashimaense*	MK685280.1 (99.82%)					x	x			U	-
	*Simplicillium* sp.	MH859771.1 (99.12%)	x				x		x		B [95]	[95]
	*Tolypocladium* sp.	KX034386.1 (100%)	x								O [96]	[97]
Pezizales	*Plectania rhytidia*	MH003435.1 (98.99%)						x	x		U	[27]
	*Pseudoplectania ericae*	MT498082.1 (99.65%)					x				U	-
	*Pyronema* sp.	MT556695.1 (100%)			x				x		O [98]	[99]
Phaeomoniellales	*Neophaeomoniella* sp.	MK646052.1 (96.14%)			x						P [100]	[101]
Pleosporales	*Alternaria alternata*	MT635274.1 (100%)		x					x		P [102]/B [103]	**[40]**
	*Alternaria* sp.	MT557456.1 (100%)		x	x		x			x	P [104]/B [103]	**[40]**
	*Angustimassarina* sp.	MN963689.1 (100%)			x						U	-
	*Camarosporium brabeji*	LN714529.1 (97.76%)							x	x	U	[105]
	*Coniothyrium carteri*	KX359604.1 (99.82%)					x	x	x		B [106]	[106]
	*Coniothyrium ferrarisianum*	MH860854.1 (100%)			x						U	[107]
	*Coniothyrium palmicola*	JX681086.1 (99.53%)						x			U	-
	*Epicoccum nigrum*	MT548679.1 (100%)			x			x	x		B **[27]**	**[40]**
	*Kalmusia* sp.	MK796143.1 (100%)					x	x			P [108]	[109]
	*Neocucurbitaria cava*	MK796144.1 (100%)		x	x				x		U	[110]
	*Neocucurbitaria* sp.	MH858303.1 (93.78%)		x	x			x	x	x	U	[110]
	*Phoma* sp.	KX815489.1 (100%)			x					x	P [111]	**[40]**
	*Preussia* sp.	MN696547.1 (100%)						x			B [112]	[113]
	*Pyrenophora biseptata*	MH864748.1 (100%)								x	P [114]	-
Sordariales	*Chaetomium globosum*	MT588864.1 (100%)		x							B [115]	[116]
	*Chaetomium *sp.	MN153902.1	x				x	x			B [117]	[118]
	*Copromyces* sp.	(100%)		x							U	-
	*Fimetariella rabenhorstii*	MN555335.1 (100%)	x	x	x						O [119]/P [120]	[121]
Xylariales	*Biscogniauxia mediterranea*	MT862330.1 (100%)	x	x	x	x	x	x	x	x	P **[12]**	**[40]**
	*Daldinia* sp.	MN341734.1 (97.83%)			x						B [122]	[123]
	*Xylaria* sp.	JQ761730.1 (99.78%)					x			x	P [124]/B [125]	[126]
Basidiomycota												
Atheliales	*Athelia arachnoidea*	MH860510.1 (100%)								x	P [127]	-

**Table 3:** Classification of fungal interactions occurring between tested endophytes and pathogens on dual-culture assay. The interaction type was classified based on endophyte/pathogen growth by “+“ for higher growth, “-“ for less growth and “0“ for equal growth in relation to control. Underlined mycelial interactions denote endophyte replacement by the pathogen.

Endophyte	*B. Mediterranea*		*D. Corticola*	
	Type of Interaction	Mycelial Interaction	Type of Interaction	Mycelial Interaction
*S. aogashimaense*	antagonism (0/-)	B	antagonism (0/-)	B
*C. carteri*	agonism (-/+)	CA2	co-antagonism (-/-)	CA2
*D. passiflorae*	agonism (-/+)	CB1	co-antagonism (-/-)	CB1
*F. rabenhorstii*	co-antagonism (-/-)	A	co-antagonism (-/-)	CA1
*F. oxysporum*	commensalism (+/0)	CA1	antagonism (0/-)	CA1
*Chaetomium* sp.	agonism (+/-)	CA1	co-antagonism (-/-)	CA1
*A. alternata*	agonism (+/-)	CA1	antagonism (0/-)	CA1
*P. olsonii*	agonism (-/+)	CB1	co-antagonism (-/-)	CA1

## Supplementary Materials

The following are available online at https://www.mdpi.com/2309-608X/6/4/287/s1, Table S1: Characterization of sampled cork oak stands; Table S2: Growth rates of cork oak endophytic fungi used for the antagonistic assays; Figure S1: Inhibition of fungal endophytes growth caused by the *B. mediterranea* and *D. corticola* in dual culture assay; Figure S2: Interaction in dual culture of *S. aogashimaense*-*B. mediterranea* (A), *S. aogashimaense*-*D. corticola* (B) and *A. alternata*- *B. mediterranea* (C) 72 h and 15 days after inoculation.

## References

[1] Gauquelin T., Michon G., Joffre R., Duponnois R., Génin D., Fady B., Dagher-Kharrat M.B., Derridj A., Slimani S., Badri W. (2018). Mediterranean forests, land use and climate change: A social-ecological perspective. Reg. Environ. Chang..

[2] FAO and Plan Bleu (2018). State of Mediterranean Forests 2018.

[3] Costa R., Lourenço A., Oliveira V., Pereira H. (2019). Chemical characterization of cork, phloem and wood from different *Quercus suber* provenances and trees. Heliyon.

[4] APCOR (2019). APCOR’s Cork Yearbook 2018/2019.

[5] Touhami I., Chirino E., Aouinti H., El Khorchani A., Elaieb M.T., Khaldi A., Nasr Z. (2020). Decline and dieback of cork oak (*Quercus suber* L.) forests in the Mediterranean basin: A case study of Kroumirie, Northwest Tunisia. J. For. Res..

[6] Giorgi F. (2006). Climate change hot-spots. Geophys. Res. Lett..

[7] Rego F.C., Rocha M.S. (2014). Climatic patterns in the Mediterranean region. Ecol. Mediterr..

[8] Shaw M.W., Osborne T.M. (2011). Geographic distribution of plant pathogens in response to climate change. Plant Pathol..

[9] La Porta N., Capretti P., Thomsen I.M., Kasanen R., Hietala A.M., Von Weissenberg K. (2008). Forest pathogens with higher damage potential due to climate change in Europe. Can. J. Plant Pathol..

[10] Elad Y., Pertot I. (2014). Climate Change Impacts on Plant Pathogens and Plant Diseases. J. Crop. Improv..

[11] Moricca S., Linaldeddu B.T., Ginetti B., Scanu B., Franceschini A., Ragazzi A. (2016). Endemic and Emerging Pathogens Threatening Cork Oak Trees: Management Options for Conserving a Unique Forest Ecosystem. Plant Dis..

[12] Linaldeddu B.T., Sirca C., Spano D., Franceschini A. (2011). Variation of endophytic cork oak-associated fungal communities in relation to plant health and water stress. For. Pathol..

[13] Linaldeddu B.T., Sirca C., Spano D., Franceschini A. (2009). Physiological responses of cork oak and holm oak to infection by fungal pathogens involved in oak decline. For. Pathol..

[14] Luque J., Pera J., Parladé J. (2008). Evaluation of fungicides for the control of *Botryosphaeria corticola* on cork oak in Catalonia (NE Spain). For. Pathol..

[15] Serrano M.S., Romero M.A., Jiménez J.J., De Vita P., Ávila A., Trapero A., Sánchez M.E. (2015). Preventive control of *Botryosphaeria* canker affecting *Quercus suber* in southern Spain. Forestry.

[16] Terhonen E., Kovalchuk A., Zarsav A., Asiegbu F.O. (2018). Biocontrol potential of forest tree endophytes. Endophytes of Forest Trees.

[17] Martín-García J., Zas R., Solla A., Woodward S., Hantula J., Vainio E.J., Mullett M., Morales-Rodríguez C., Vannini A., Martínez-Álvarez P. (2019). Environmentally friendly methods for controlling pine pitch canker. Plant Pathol..

[18] Campanile G., Ruscelli A., Luisi N. (2007). Antagonistic activity of endophytic fungi towards *Diplodia corticola* assessed by in vitro and in planta tests. Eur. J. Plant Pathol..

[19] Linaldeddu B.T., Maddau L., Franceschini A. (2005). Preliminary in vitro investigation on the interactions among endophytic fungi isolated from *Quercus* spp.. IOBC WPRS Bull..

[20] Maddau L., Cabras A., Franceschini A., Linaldeddu B.T., Crobu S., Roggio T., Pagnozzi D. (2009). Occurrence and characterization of peptaibols from *Trichoderma citrinoviride*, an endophytic fungus of cork oak, using electrospray ionization quadrupole time-of-flight mass spectrometry. Microbiology.

[21] Karami J., Kavosi M.R., Babanezhad M., Kiapasha K. (2018). Integrated management of the charcoal disease by silviculture, chemical and biological methods in forest parks. J. Sustain. For..

[22] Martins F., Pereira J.A., Bota P., Bento A., Baptista P. (2016). Fungal endophyte communities in above- and belowground olive tree organs and the effect of season and geographic location on their structures. Fungal Ecol..

[23] Costa D., Tavares R., Baptista P., Lino-Neto T. (2018). Diversity of fungal endophytic community in *Quercus suber* L. under different climate scenarios. Rev. Ciências Agrárias.

[24] White T.J., Bruns T., Lee SJ W.T., Taylor J.L. (1990). Amplification and direct sequencing of fungal ribosomal rna genes for phylogenetics. PCR Protoc. Guid. Methods Appl..

[25] Nilsson R.H., Larsson K.-H., Taylor A.F.S., Bengtsson-Palme J., Jeppesen T.S., Schigel D., Kennedy P., Picard K., Glöckner F.O., Tedersoo L. (2019). The UNITE database for molecular identification of fungi: Handling dark taxa and parallel taxonomic classifications. Nucleic Acids Res..

[26] Hardoim P.R., Van Overbeek L.S., Berg G., Pirttilä A.M., Compant S., Campisano A., Döring M., Sessitsch A. (2015). The Hidden World within Plants: Ecological and Evolutionary Considerations for Defining Functioning of Microbial Endophytes. Microbiol. Mol. Biol. Rev..

[27] Gomes T., Pereira J.A., Lino-Neto T., Bennett A.E., Baptista P. (2019). Bacterial disease induced changes in fungal communities of olive tree twigs depend on host genotype. Sci. Rep..

[28] Schneider C.A., Rasband W.S., Eliceiri K.W. (2012). NIH Image to ImageJ: 25 years of image analysis. Nat. Methods.

[29] Tuininga A., Dighton J., White J.P.O. (2005). Interspecific interaction terminology: From mycology to general ecology. The Fungal Community: Its Organization and Role in the Ecosystem.

[30] Badalyan S.M., Innocenti G., Garibyan N.G. (2002). Antagonistic Activity of Xylotrophic Mushrooms against Pathogenic Fungi of Cereals in Dual Culture. Phytopathol. Mediterr..

[31] Dennis C., Webster J. (1971). Antagonistic properties of species-groups of Trichoderma. Trans. Br. Mycol. Soc..

[32] Wu B., Hussain M., Zhang W., Stadler M., Liu X., Xiang M. (2019). Current insights into fungal species diversity and perspective on naming the environmental DNA sequences of fungi. Mycology.

[33] Dissanayake A.J., Purahong W., Wubet T., Hyde K.D., Zhang W., Xu H., Zhang G., Fu C., Liu M., Xing Q. (2018). Direct comparison of culture-dependent and culture-independent molecular approaches reveal the diversity of fungal endophytic communities in stems of grapevine (*Vitis vinifera*). Fungal Divers..

[34] Gomes T., Pereira J.A., Benhadi J., Lino-Neto T., Baptista P. (2018). Endophytic and Epiphytic Phyllosphere Fungal Communities Are Shaped by Different Environmental Factors in a Mediterranean Ecosystem. Microb. Ecol..

[35] Bragança H., Machado H., Inácio L., Henriques J., Diogo E., Moreira C. (2013). Detecção de agentes potencialmente patogénicos em sobreiro e azinheira. Abstracts of the Congresso Florestal Nacional.

[36] Smahi H., Belhoucine-Guezouli L., Bouhraoua R.T., Franceschini A., Linaldeddu B.T. (2018). First Report of Branch Canker and Dieback Caused by *Cryphonectria naterciae* on *Quercus suber* in Algeria. Plant Dis..

[37] Luque J., Parladé J., Pera J. (2000). Pathogenicity of fungi isolated from *Quercus suber* in Catalonia (NE Spain). For. Pathol..

[38] Bragança H., Neno J., Henriques J., Diogo E., Alves A. (2016). First Report of *Diplodia quercivora* Causing Dieback on *Quercus suber* and in Europe. Plant Dis..

[39] Ragazzi A., Turco E., Marianelli L., Dellavalle I., Moricca S. (2007). Disease gradient of the anthracnose agent *Apiognomonia quercina* in a natural oak stand. Phytopathol. Mediterr..

[40] Franceschini A., Linaldeddu B.T., Marras F. (2005). Occurrence and distribution of fungal endophytes in declining cork oak forests in Sardinia (Italy). IOBC WPRS Bull..

[41] Ferreira S.L., Stauder C.M., Martin D., Kasson M.T. (2020). Morphological and Phylogenetic Resolution of *Diplodia corticola* and *D. quercivora*, Emerging Canker Pathogens of Oak (*Quercus* spp.), in the United States. Plant Dis..

[42] Linaldeddu B.T., Franceschini A., Alves A., Phillips A.J.L. (2013). *Diplodia quercivora* sp. nov.: A new species of *Diplodia* found on declining *Quercus canariensis* trees in Tunisia. Mycologia.

[43] Bakker P.A., Pieterse C.M., De Jonge R., Berendsen R.L. (2018). The Soil-Borne Legacy. Cell.

[44] Liu H., Macdonald C.A., Cook J., Anderson I.C., Singh B.K. (2019). An Ecological Loop: Host Microbiomes across Multitrophic Interactions. Trends Ecol. Evol..

[45] Lombardi N., Vitale S., Turrà D., Reverberi M., Fanelli C., Vinale F., Marra R., Ruocco M., Pascale A., D’Errico G. (2018). Root Exudates of Stressed Plants Stimulate and Attract Trichoderma Soil Fungi. Mol. Plant-Microbe Interact..

[46] Crous P., Wingfield M.J., Guarro J., Cheewangkoon R., Van Der Bank M., Swart W.J., Stchigel A.M., Cano-Lira J.F., Roux J., Madrid H. (2013). Fungal Planet description sheets: 154–213. Persoonia Mol. Phylogeny Evol. Fungi.

[47] Rahi P., Vyas P., Sharma S., Gulati A., Gulati A. (2009). Plant growth promoting potential of the fungus *Discosia* sp. FIHB 571 from tea rhizosphere tested on chickpea, maize and pea. Indian J. Microbiol..

[48] Szink I., Davis E.L., Ricks K.D., Koide R.T. (2016). New evidence for broad trophic status of leaf endophytic fungi of *Quercus gambelii*. Fungal Ecol..

[49] Smahi H., Belhoucine-Guezouli L., Berraf-Tebbal A., Chouih S., Arkam M., Franceschini A., Linaldeddu B.T., Phillips A.J.L. (2017). Molecular characterization and pathogenicity of *Diplodia corticola* and other Botryosphaeriaceae species associated with canker and dieback of *Quercus suber* in Algeria. Mycosphere.

[50] Li H., Li Z., Ruan G., Yu Y., Liu X. (2016). Asymmetric reduction of acetophenone into R-(+)-1-phenylethanol by endophytic fungus *Neofusicoccum parvum* BYEF07 isolated from *Illicium verum*. Biochem. Biophys. Res. Commun..

[51] Barbosa M.A.G., Rehn K.G., Menezes M., Mariano R.D.L.R. (2001). Antagonism of *Trichoderma* species on *Cladosporium herbarum* and their enzimatic characterization. Braz. J. Microbiol..

[52] Larran S., Perelló A., Simón M.R., Moreno V. (2007). The endophytic fungi from wheat (*Triticum aestivum* L.). World J. Microbiol. Biotechnol..

[53] Oliveira R.R., Aguiar R.L., Tessmann D.J., Nunes W.M.C., Santos A.F., Vida J.B. (2014). First Report of Leaf Spot Caused by *Cladosporium perangustum* on *Syagrus oleracea* in Brazil. Plant Dis..

[54] Ashkezari S.J., Fotouhifar K.-B. (2017). Diversity of endophytic fungi of common yew (*Taxus baccata* L.) in Iran. Mycol. Prog..

[55] Jones D.A., Thomas C.M., Hammond-Kosack K.E., Balint-Kurti P.J., Jones J.D.G. (1994). Isolation of the tomato Cf-9 gene for resistance to *Cladosporium fulvum* by transposon tagging. Science.

[56] Wang X., Gul W., Taráwneh A.H., Gao J., Wedge D.E., Rosa L.H., Cutler H.G., Cutler S.J. (2013). Antifungal Activity against Plant Pathogens of Metabolites from the Endophytic Fungus *Cladosporium cladosporioides*. J. Agric. Food Chem..

[57] Hamayun M., Khan S.A., Ahmad N., Tang D.-S., Kang S.-M., Na C.-I., Sohn E.-Y., Hwang Y.-H., Shin D.-H., Lee B.-H. (2009). *Cladosporium sphaerospermum* as a new plant growth-promoting endophyte from the roots of *Glycine max* (L.) Merr. World J. Microbiol. Biotechnol..

[58] Pascoe I., (Maher) P.M., Smith I., Dinh S.-Q., Edwards J. (2018). *Caliciopsis pleomorpha* sp. nov. (Ascomycota: Coryneliales) causing a severe canker disease of *Eucalyptus cladocalyx* and other eucalypt species in Australia. Fungal Syst. Evol..

[59] Panteleev S.V., Baranov O.Y., Rubel I.E., Yarmolovich V.A., Dishuk N.G., Seredich M.O. (2016). Diseases of Container-Grown Conifers in the Nurseries of Mogilev Area According to Molecular Phytopathological Survey. Proceedings of BSTU.

[60] Alidadi A., Kowsari M., Javan-Nikkhah M., Jouzani G.R.S., Rastaghi M.E. (2019). New pathogenic and endophytic fungal species associated with Persian oak in Iran. Eur. J. Plant Pathol..

[61] Lawrence D.P., Travadon R., Pouzoulet J., Rolshausen P.E., Wilcox W.F., Baumgartner K. (2016). Characterization of *Cytospora* isolates from wood cankers of declining grapevine in North America, with the descriptions of two newCytosporaspecies. Plant Pathol..

[62] Jiang N., Fan X.-L., Crous P.W., Tian C. (2019). Species of *Dendrostoma* (Erythrogloeaceae, Diaporthales) associated with chestnut and oak canker diseases in China. MycoKeys.

[63] Li H., Yu S., Tang W., Miao M., Liu Y. (2019). First Report of *Diaporthe passiflorae* and *Diaporthe nobilis* Causing a Postharvest Kiwifruit Rot in Sichuan Province, China. Plant Dis..

[64] Elfar K., Torres R., Díaz G.A., Latorre B.A. (2013). Characterization of *Diaporthe australafricana* and Diaporthe spp. Associated with Stem Canker of Blueberry in Chile. Plant Dis..

[65] Gomes R.R., Glienke C., Videira S.I.R., Lombard L., Groenewald J.Z., Crous P. (2013). *Diaporthe*: A genus of endophytic, saprobic and plant pathogenic fungi. Pers. Mol. Phylogeny Evol. Fungi.

[66] Venkatasubbaiah P., Chilton W.S. (1991). Toxins Produced by the Dogwood Anthracnose Fungus *Discula* sp.. J. Nat. Prod..

[67] Ganley R.J., Brunsfeld S.J., Newcombe G. (2004). A community of unknown, endophytic fungi in western white pine. Proc. Natl. Acad. Sci. USA.

[68] Castoria R., De Curtis F., Lima G., Caputo L., Pacifico S., De Cicco V. (2001). *Aureobasidium pullulans* (LS-30) an antagonist of postharvest pathogens of fruits: Study on its modes of action. Postharvest Biol. Technol..

[69] Wachowska U., Głowacka K. (2014). Antagonistic interactions between *Aureobasidium pullulans* and *Fusarium culmorum*, a fungal pathogen of winter wheat. BioControl.

[70] Bardas G.A., Tzelepis G.D., Lotos L., Karaoglanidis G.S. (2009). First Report of *Penicillium glabrum* Causing Fruit Rot of Pomegranate (*Punica granatum*) in Greece. Plant Dis..

[71] Hammerschmidt L., Wray V., Lin W., Kamilova E., Proksch P., Aly A.H. (2012). New styrylpyrones from the fungal endophyte *Penicillium glabrum* isolated from *Punica granatum*. Phytochem. Lett..

[72] Demirci E., Dane E., Eken C. (2011). In vitro antagonistic activity of fungi isolated from sclerotia on potato tubers against *Rhizoctonia solani*. Turk. J. Biol..

[73] Vega F.E., Posada F., Peterson S.W., Gianfagna T.J., Chaves F. (2006). *Penicillium* species endophytic in coffee plants and ochratoxin A production. Mycologia.

[74] Stierle A.A., Stierle D.B. (2000). Bioactive Compounds from four Endophytic *Penicillium* sp. of a Northwest Pacific Yew Tree. Studies in Natural Products Chemistry.

[75] Whetzel H.H., Wolf F.A. (1945). The Cup Fungus, *Ciboria carunculoides*, Pathogenic on Mulberry Fruits. Mycologia.

[76] Kehr R.D. (1991). *Pezicula* canker of *Quercus rubra* L., caused by *Pezicula cinnamomea* (DC.) Sacc. I. Symptoms and pathogenesis. Eur. J. For. Pathol..

[77] Bissegger M., Sieber T.N. (1994). Assemblages of Endophytic Fungi in Coppice Shoots of *Castanea sativa*. Mycologia.

[78] Chen C., Verkley G.J.M., Sun G., Groenewald J.Z., Crous P.W. (2016). Redefining common endophytes and plant pathogens in *Neofabraea*, *Pezicula*, and related genera. Fungal Biol..

[79] Yuan Z., Verkley G.J.M. (2015). *Pezicula neosporulosa* sp. nov. (Helotiales, Ascomycota), an endophytic fungus associated with *Abies* spp. in China and Europe. Mycoscience.

[80] McMullin D.R., Green B.D., Prince N.C., Tanney J.B., Miller J.D. (2017). Natural Products of *Picea* Endophytes from the Acadian Forest. J. Nat. Prod..

[81] Liu K.H., Ding X., Deng B.W., Chen W. (2009). Isolation and characterization of endophytic taxol-producing fungi from *Taxus chinensis*. J. Ind. Microbiol. Biotechnol..

[82] Schulz B., Sucker J., Aust H.J., Krohn K., Ludewig K., Jones P.G., Döring D. (1995). Biologically active secondary metabolites of endophytic *Pezicula* species. Mycol. Res..

[83] Xue A.G. (2003). Biological Control of Pathogens Causing Root Rot Complex in Field Pea Using *Clonostachys rosea* Strain ACM941. Phytopathology.

[84] Cannon P.F., Simmons C.M. (2002). Diversity and host preference of leaf endophytic fungi in the Iwokrama Forest Reserve, Guyana. Mycologia.

[85] Madar Z., Kimchi M., Solel Z. (1996). *Fusarium* canker of Italian cypress. Eur. J. For. Pathol..

[86] Manici L.M., Kelderer M., Franke-Whittle I.H., Rühmer T., Baab G., Nicoletti F., Caputo F., Topp A., Insam H., Naef A. (2013). Relationship between root-endophytic microbial communities and replant disease in specialized apple growing areas in Europe. Appl. Soil Ecol..

[87] Di Pietro A., García-Maceira F.I., Méglecz E., Roncero M.I.G. (2001). A MAP kinase of the vascular wilt fungus *Fusarium oxysporum* is essential for root penetration and pathogenesis. Mol. Microbiol..

[88] Bolwerk A., Lagopodi A.L., Lugtenberg B.J.J., Bloemberg G.V. (2005). Visualization of Interactions Between a Pathogenic and a Beneficial *Fusarium* Strain During Biocontrol of Tomato Foot and Root Rot. Mol. Plant-Microbe Interact..

[89] Kour A., Shawl A.S., Rehman S., Sultan P., Qazi P.H., Suden P., Khajuria R.K., Verma V. (2008). Isolation and identification of an endophytic strain of *Fusarium oxysporum* producing podophyllotoxin from *Juniperus recurva*. World J. Microbiol. Biotechnol..

[90] Fernández-Silva F., Capilla J., Mayayo E., Sutton D., Guarro J. (2014). In VitroEvaluation of Antifungal Drug Combinations against *Sarocladium* (*Acremonium*) *kiliense*, an Opportunistic Emergent Fungus Resistant to Antifungal Therapies. Antimicrob. Agents Chemother..

[91] Campos L.A. (2017). Caracterização de Leveduras Promotoras do Crescimento de Plantas.

[92] Yuan W.H., Jiang N., Dong C.H., Wei Z.W., Wu H.K., Chen C.F., Zhao Y.X., Zhou S.L., Zhang M.M., Zheng W.F. (2013). Lasiodiplodin analogues from the endophytic fungus Sarocladium kiliense. Chem. Pharm. Bull..

[93] Tschen J.S.M., Chen L.L., Hsieh S.T., Wu T.S. (1997). Isolation and phytotoxic effects of helvolic acid from plant pathogenic fungus Sarocladium oryzae. Bot. Bull. Acad. Sin..

[94] Potshangbam M., Indira S., Sahoo D., Strobel G.A. (2017). Functional Characterization of Endophytic Fungal Community Associated with *Oryza sativa* L. and *Zea mays* L.. Front. Microbiol..

[95] Gomes A.A.M., Pinho D.B., Cardeal Z.D.L., Menezes H.C., De Queiroz M.V., Pereira O.L. (2018). *Simplicillium coffeanum*, a new endophytic species from Brazilian coffee plants, emitting antimicrobial volatiles. Phytotaxa.

[96] Herrero N., Zabalgogeazcoa I. (2011). Mycoviruses infecting the endophytic and entomopathogenic fungus *Tolypocladium cylindrosporum*. Virus Res..

[97] Hanada R.E., Pomella A.W.V., Costa H.S., Bezerra J.L., Loguercio L.L., Pereira J.O. (2010). Endophytic fungal diversity in *Theobroma cacao* (cacao) and *T. grandiflorum* (cupuaçu) trees and their potential for growth promotion and biocontrol of black-pod disease. Fungal Biol..

[98] Deng Z., Li C., Luo D., Teng P., Guo Z., Tu X., Zou K., Gong D. (2017). A new cinnamic acid derivative from plant-derived endophytic fungus *Pyronema* sp.. Nat. Prod. Res..

[99] Botella L., Diez J.J. (2011). Phylogenic diversity of fungal endophytes in Spanish stands of *Pinus halepensis*. Fungal Divers..

[100] Ferreira A.B.M., Leite L.G., Hernandes J.L., Harakava R., Padovani C.R., Bueno C.J. (2018). Colonization of vines by Petri disease fungi, susceptibility of rootstocks to *Phaeomoniella chlamydospora* and their disinfection. Arquivos Instituto Biológico.

[101] Lacerda L.T., Gusmão L.F.P., Rodrigues A. (2018). Diversity of endophytic fungi in *Eucalyptus microcorys* assessed by complementary isolation methods. Mycol. Prog..

[102] Peever T.L., Ibañez A., Akimitsu K., Timmer L.W. (2002). Worldwide Phylogeography of the Citrus Brown Spot Pathogen, *Alternaria alternata*. Phytopathology.

[103] Soltani J., Moghaddam M.S.H. (2014). Antiproliferative, Antifungal, and Antibacterial Activities of Endophytic *Alternaria* Species from Cupressaceae. Curr. Microbiol..

[104] Thomma B.P.H.J. (2003). *Alternaria* spp.: From general saprophyte to specific parasite. Mol. Plant Pathol..

[105] Jinu M., Jayabaskaran C. (2015). Diversity and anticancer activity of endophytic fungi associated with the medicinal plant Saraca asoca. Curr. Res. Environ. Appl. Mycol..

[106] Qadri M., Rajput R., Abdin M.Z., Vishwakarma R.A., Riyaz-Ul-Hassan S. (2014). Diversity, Molecular Phylogeny, and Bioactive Potential of Fungal Endophytes Associated with the Himalayan Blue Pine (*Pinus wallichiana*). Microb. Ecol..

[107] Ibrahim A., Sørensen D., Jenkins H.A., Ejim L., Capretta A., Sumarah M.W. (2017). Epoxynemanione A, nemanifuranones A–F, and nemanilactones A–C, from *Nemania serpens*, an endophytic fungus isolated from Riesling grapevines. Phytochemistry.

[108] Abed-Ashtiani F., Narmani A., Arzanlou M. (2019). Analysis of *Kalmusia variispora* associated with grapevine decline in Iran. Eur. J. Plant Pathol..

[109] Ghobad-Nejhad M., Asgari B., Dokhaharani S.C. (2017). Notes on some endophytic fungi isolated from *Quercus brantii* in Dena Region of Kohgiluyeh and Boyer-Ahmad Province. Iran. Mycol. Iran..

[110] Kwaśna H., Szewczyk W., Behnke-Borowczyk J. (2015). Fungal root endophytes of *Quercus robur* subjected to flooding. For. Pathol..

[111] Strobel G., Singh S.K., Riyaz-Ul-Hassan S., Mitchell A., Geary B., Sears J. (2011). An endophytic/pathogenic Phoma sp. from creosote bush producing biologically active volatile compounds having fuel potential. FEMS Microbiol. Lett..

[112] Weber H.A., Gloer J.B. (1991). The preussomerins: Novel antifungal metabolites from the coprophilous fungus *Preussia isomera* Cain. J. Org. Chem..

[113] Pulina M.A., Linaldeddu B.T., Franceschini A. Topoclimats et communautés des champignons endophytiques dans des bois de chênes-lièges dépéris et non dépéris en Sardaigne (Italie). Proceddings of XIXe COLLOQUE INTERNATIONAL DE CLIMATOLOGIE.

[114] Summerell B.A., McGovern R.E.W. (2017). Diseases of Proteaceae. Handbook of Plant Disease Management.

[115] Zhao S.-S., Zhang Y.-Y., Yan W., Cao L.-L., Xiao Y., Ye Y.-H. (2016). *Chaetomium globosum* CDW7, a potential biological control strain and its antifungal metabolites. FEMS Microbiol. Lett..

[116] Qin C., Tao J., Liu T., Liu Y., Xiao N., Li T., Gu Y., Yin H., Meng D. (2019). Responses of phyllosphere microbiota and plant health to application of two different biocontrol agents. AMB Express.

[117] Tveit M., Wood R.K.S. (1955). The control of *Fusarium* blight in oat seedlings with antagonistic species of chaetomium. Ann. Appl. Biol..

[118] Fisher P., Petrini O., Petrini L. (1991). Endophytic ascomycetes and deuteromycetes in roots of Pinus sylvestris. Nov. Hedwig..

[119] Deng L., Niu S., Liu X., Che Y., Li E. (2013). Coniochaetones E–I, new 4H-chromen-4-one derivatives from the Cordyceps-colonizing fungus *Fimetariella* sp. Fitoterapia.

[120] Bashiri S., Abdollahzadeh J., Di Lecce R., Alioto D., Górecki M., Pescitelli G., Masi M., Evidente A. (2020). Rabenchromenone and Rabenzophenone, Phytotoxic Tetrasubstituted Chromenone and Hexasubstituted Benzophenone Constituents Produced by the Oak-Decline-Associated Fungus *Fimetariella rabenhorstii*. J. Nat. Prod..

[121] Tao M.H., Li D.-L., Zhang W.-M., Tan J.-W., Wei X.-Y. (2011). [Study on the chemical constituents of endophytic fungus *Fimetariella rabenhorstii* isolated from *Aquilaria sinensis*]. Zhong Yao Cai.

[122] Liarzi O., Bar E., Lewinsohn E., Ezra D. (2016). Use of the Endophytic Fungus *Daldinia* cf. concentrica and Its Volatiles as Bio-Control Agents. PLoS ONE.

[123] Higginbotham S.J., Arnold A.E., Ibañez A., Spadafora C., Coley P.D., Kursar T.A. (2013). Bioactivity of Fungal Endophytes as a Function of Endophyte Taxonomy and the Taxonomy and Distribution of Their Host Plants. PLoS ONE.

[124] Ko W.H., Kunimoto R.K. (1991). Quick decline of macadamia trees: Association with *Xylaria arbuscula*. Plant Pathol..

[125] Park J.H., Choi G.J., Lee H.B., Kim K.M., Jung H.S., Lee S.W., Jang K.S., Cho K.Y., Kim J.C. (2005). Griseofulvin from *Xylaria* sp. Strain F0010, an endophytic fungus of *Abies holophylla* and its antifungal activity against plant pathogenic fungi. J. Microbiol. Biotechnol..

[126] Liu X., Dong M., Chen X., Jiang M., Lv X., Yan G. (2007). Antioxidant activity and phenolics of an endophytic *Xylaria* sp. from *Ginkgo biloba*. Food Chem..

[127] Adams G.C., Kropp B.R. (1996). *Athelia arachnoidea*, the sexual state of *Rhizoctonia carotae*, a pathogen of carrot in cold storage. Mycologia.

[128] Kotasthane A.S., Agrawal T., Kushwah R., Rahatkar O.V. (2014). In-vitro antagonism of *Trichoderma* spp. against *Sclerotium rolfsii* and *Rhizoctonia solani* and their response towards growth of cucumber, bottle gourd and bitter gourd. Eur. J. Plant Pathol..

[129] Ujor V.C., Adukwu E.C., Okonkwo C.C. (2018). Fungal wars: The underlying molecular repertoires of combating mycelia. Fungal Biol..

[130] Escano-Calderón C., Rotem N., Harris R., Vela-Corcía D., Levy M. (2019). *Pseudozyma aphidis* activates reactive oxygen species production, programmed cell death and morphological alterations in the necrotrophic fungusBotrytis cinerea. Mol. Plant Pathol..

[131] Fatima N., Muhammad S.A., Khan I., Qazi M.A., Shahzadi I., Mumtaz A., Hashmi M.A., Khan A.K., Ismail T. (2016). *Chaetomium* endophytes: A repository of pharmacologically active metabolites. Acta Physiol. Plant..

[132] Yang N. (2017). Secondary Metabolites Isolated from *Coniothyrium* Species. Nat. Prod. J..

[133] Tanapichatsakul C., Monggoot S., Gentekaki E., Pripdeevech P. (2018). Antibacterial and Antioxidant Metabolites of *Diaporthe* spp. Isolated from Flowers of *Melodorum fruticosum*. Curr. Microbiol..

[134] Gandía M., Monge A., Garrigues S., Orozco H., Giner-Llorca M., Marcos J.F., Manzanares P. (2020). Novel insights in the production, activity and protective effect of *Penicillium expansum* antifungal proteins. Int. J. Biol. Macromol..

[135] Chen R.-S., Huang C.-C., Li J.-C., Tsay J.-G. (2017). Evaluation of characteristics of *Simplicillium lanosoniveum* on pathogenicity to aphids and in vitro antifungal potency against plant pathogenic fungi. Int. J. Environ. Agric. Res..

[136] Caesar L.K., Cech N.B. (2019). Synergy and antagonism in natural product extracts: When 1 + 1 does not equal 2. Nat. Prod. Rep..

[137] Shin T.S., Yu N.H., Lee J., Choi G.J., Kim J.-C., Shin C.S. (2017). Development of a Biofungicide Using a Mycoparasitic Fungus *Simplicillium lamellicola* BCP and Its Control Efficacy against Gray Mold Diseases of Tomato and Ginseng. Plant Pathol. J..

[138] Fravel D., Olivain C., Alabouvette C. (2003). *Fusarium oxysporum* and its biocontrol. New Phytol..

[139] Gauthier N.A.W., Robertson C.L., Chanda A.K., Schneider R.W. (2012). Effects of *Simplicillium lanosoniveum* on *Phakopsora pachyrhizi*, the Soybean Rust Pathogen, and Its Use as a Biological Control Agent. Phytopathology.

