# Supplementary material for: Cork Oak Endophytic Fungi as Potential Biocontrol Agents against Biscogniauxia mediterranea and Diplodia corticola
Source: J Fungi (Basel). 2020 Nov 14;6(4):287. doi: 10.3390/jof6040287 (PMC7711870; doi:10.3390/jof6040287)
Supplement: Supplementary file 1 [file jof-06-00287-s001.pdf]

Table S1. Characterization of sampled cork oak stands. Temperatures and precipitation values from the 10 years previous to sampling collection are displayed (Tmax is the medium maximum temperature of the warmest month; Tmin is the medium minimum temperature of the coldest month; P annual is the annual precipitation).

Location	Tmax/Tmin (°C)	P annual (mm)	Cork oak stand	GPS coordinates	Soil granulometry	Vegetation cover
**Peneda-Gerês**	26.7/3.0	1384.6	PG-ER	41° 42' N<br>8° 6' W	Sandy loam	*Dryopteris* sp.<br>*Pyrus communis*<br>*Geranium robertianum*<br>*Pterospartum tridentatum*<br>*Lithodora prostrata*<br>*Erica* sp.<br>*Genista falcata*<br>*Arbutus unedo*
			PG-RC	41° 45' N<br>8° 1' W	Loamy sand	*Pterospartum tridentatum*<br>*Erica scoparia*<br>*Dryopteris dilatata*<br>*Cytisus striatus*
**Limãos**	29.4/2.3	994.7	LI	41° 31' N<br>6° 49' W	Sandy clay loam	*Cytisus multiflorus*<br>*Cytisus scoparius*<br>*Daphne gnidium*<br>*Cistus ladanifer*<br>*Cistus salvifolius*
**Gavião**	30.5/6.7	844.3	GV	39° 27' N<br>7° 55' W	Loam	*Cistus salvifolius*<br>*Cistus ladanifer*<br>*Cistus crispus*<br>*Ulex airensis*<br>*Senecio jacobaea*<br>*Lavandula stoechas*<br>*Lavandula pedunculata*
**Alcobaça**	24.7/8.0	820.7	AL	39° 27' N<br>9° 2' W	Sandy clay loam	*Cistus salviifolius*<br>*Cistus crispus*<br>*Myrtus communis*<br>*Ulex europaeus*<br>*Rubia peregrina*<br>*Rubus ulmifolius*<br>*Rosa sempervirens*<br>*Anagallis monelli,*<br>*Erica* sp.
**Grândola**	30.3/7.3	739.7	GR	38° 11' N<br>8° 37' W	Sandy loam	*Cistus psilosepalus*<br>*Cistus salvifolius*<br>*Cynara cardunculus*<br>*Cirsium vulgare*<br>*Taraxacum officinalis*<br>*Asparagus aphyllus*<br>*Cystus ladanifer*
**Herdade Contenda**	32.5/4.8	636.6	HC-CT	38° 2' N<br>7° 0.5' W	Loam	*Lavandula stoechas*<br>*Cistus ladanifer*
			HC-MA	38° 2' N<br>7° 1.9' W	Loam	*Lavandula stoechas*<br>*Cistus ladanifer*

Table S2. Growth rates of cork oak endophytic fungi used for the antagonistic assays.

**Fungal species**	**Growth rate (cm^2/h)**
Diplodia corticola	0.71
Biscogniauxia mediterranea	0.34
Simplicillium aogashimaense	0.04
Coniothyrium carteri	0.04
Diaporthe passiflorae	0.04
Fimetariella rabenhorstii	0.13
Fusarium oxysporum	0.25
Chaetomium sp.	0.26
Alternaria alternata	0.30
Penicillium olsonii	0.05

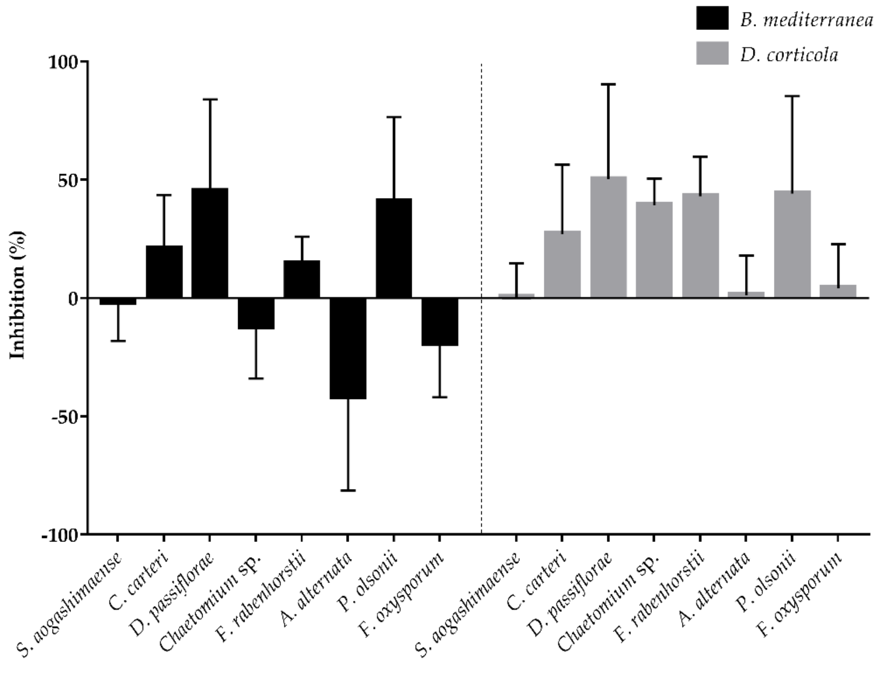

Figure S1. Inhibition of fungal endophytes growth caused by the *B. mediterranea* and *D. corticola* in dual culture assay. Negative values indicate that the area of the plate covered by the interacting pathogen was higher than that of the control.

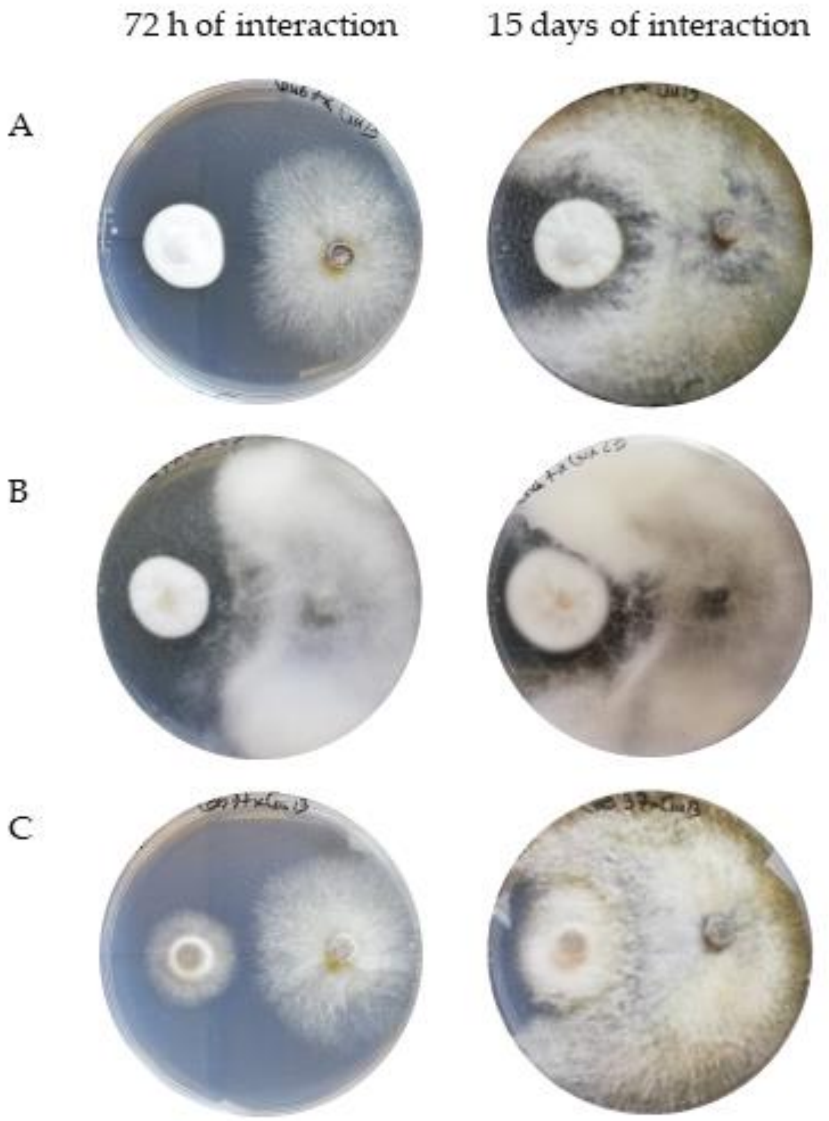

Figure S2. Interaction in dual culture of *S. aogashimaense-B. mediterranea* (A), *S. aogashimaense-D. corticola* (B) and *A. alternata- B. mediterranea* (C) 72 h and 15 days after inoculation.
